# Hepatic steatosis: a risk factor for increased COVID-19 prevalence and severity—a computed tomography study

**DOI:** 10.1186/s43066-021-00131-6

**Published:** 2021-07-12

**Authors:** Asmaa Ali, Mona Hasan, Shaimaa Hamed, Amir Elhamy

**Affiliations:** Theodor Bilharz Institute, Kornish Elnil, Embaba, Giza, Egypt

**Keywords:** Fatty liver, Computed tomography, COVID-19

## Abstract

**Background:**

Around 25% of the world population was affected by the metabolic-related fatty liver disorder. Hepatic steatosis is frequently observed in conjunction with hypertension, obesity comorbidities, and diabetes. We evaluate the hepatic steatosis frequency found in chest CT exams of COVID-19-positive cases compared to non-infected controls and evaluate the related increased prevalence and severity of COVID.

**Results:**

Our research includes 355 subjects, 158 with positive PCR for COVID-19 (case group) and 197 with negative PCR and negative CT chest (control group). The mean age in the positive group was 50.6 ± 16 years, and in the control, it was 41.3 ± 16 years (p < 0.001). Our study consists of 321 men (90.5%) and 34 women (9.5%). The number of males in both cases and control groups was greater. In the case group, 93% men vs. 6.9% women, while in controls, 88.3% men vs.11.6% women, p < 0.001. CT revealed normal results in 55.5% of individuals (i.e., CORADs 1) and abnormal findings in 45.5% of participants (i.e., CORADs 2–5). In abnormal scan, CO-RADs 2 was 13.92%, while CO-RADs 3–4 were 20.89% of cases. CO-RADs 5 comprised 65.19% of all cases. Approximately 42.6% of cases had severe disease (CT score ≥ 20), all of them were CO-RADs 5. The PCR-positive class had a greater prevalence of hepatic steatosis than controls (28.5% vs.12.2%, p < 0.001). CO-RADs 2 represented 11.1%, CO-RADs 3–4 represented 15.6%, and CO-RADs 5 represented 73.3% in the hepatic steatosis cases. The mean hepatic attenuation value in the case group was 46.79 ± 12.68 and in the control group 53.34 ± 10.28 (p < 0.001). When comparing patients with a higher severity score (CT score ≥ 20) to those with non-severe pneumonia, it was discovered that hepatic steatosis is more prevalent (73.2% vs. 26.8%).

**Conclusions:**

Steatosis was shown to be substantially more prevalent in COVID-19-positive individuals. There is a relation among metabolic syndrome, steatosis of the liver, and obesity, as well as the COVID-19 severity.

## Key points

- High-resolution computed tomography aids clinicians in evaluating lung affection in COVID-positive cases.
- Fatty liver and obesity are rising globally.
- Fatty liver and metabolic syndrome are significant predisposing parameters for COVID-19 infection and increase disease’s severity.

## Background

The World Health Organization declared coronavirus disease 2019 (COVID-19) as a pandemic on March 11, 2020 [1]. By May 2021, around 165,772,430 reported cases and 3,437,545 mortalities had occurred (https://www.who.org). COVID-19 is symptomatized by fever and dry cough, and the infection is diagnosed by a real-time reverse transcription-polymerase chain reaction (RT-PCR) test [2, 3]. Due to the rise in global cases, other symptoms like constipation, diarrhea, abdominal pain, and vomiting have risen; these are associated with abnormal renal and liver functions, and D-Dimer levels [4, 5]

COVID-19 can impact other organs besides the respiratory system, like the cardiovascular system, kidneys, liver, and coagulation system [6–10].

Diabetes, age, metabolic syndrome, hypertension, and obesity are all risk parameters for severe/critical illness and death [11–13].

People with obesity and type 2 diabetes are at greater risk for non-alcoholic fatty liver disease NAFLD, that worsen these disorders. It has been linked to an inflammatory response (increased neutrophil-to-lymphocyte ratio [NLR]) and subsequent poor outcomes in COVID-19-infected cases [14].

NAFLD has risen over the last two decades, affecting around 24% of the individual [15, 16]. NAFLD is a complex process with hepatic and extrahepatic pathophysiology and clinical symptoms. It leads to ectopic fatty substrate deposits in the liver, ranging from simple steatosis without inflammation to steatohepatitis, which causes cirrhosis and fibrosis [15].

CT affects the care of COVID-19 individuals because it aids in the early discovery and diagnosis, particularly in cases when the RT-PCR result is false-negative [16].

COVID-19 chest CT results are typically multifocal bilateral, mostly peripheral subpleural round, ground-glass opacities with or without patchy consolidations affecting mostly the posterior lower lobes [17]. Additionally, airway changes, reversed halo sign, and crazy paving patterns can be detected [18]. The Radiological Society of North America (RSNA) defined four categories on reporting chest CT findings in COVID-19 pneumonia: (1) typical features that are usually reported in COVID-19, (2) indeterminate features that are not characteristic of COVID-19 pneumonia, (3) atypical features that are uncommon in COVID-19 pneumonia but can occur with other infections, and (4) negative for lung inflammation with no lung results denoting infection. Chest CT may be negative in the early stages of COVID-19 infection [19].

We frequently include the upper abdomen in the regular CT scan of the chest conducted to assess cases with COVID-19 pneumonia, so that most of the liver and spleen can be viewed and examined [20].

The regular liver appears slightly more attenuated on non-contrast CT than the blood and spleen, and the intrahepatic arteries present as hypo-attenuated structures. Although histopathological analysis and liver biopsy are the gold measure for determining hepatic steatosis, they are invasive procedures. As a result, numerous studies have examined non-invasive alternatives to liver biopsy utilizing CT imaging [21].

Unenhanced CT liver attenuation alone is highly specific for moderate to severe hepatic steatosis, obviating the requirement for verification by biopsy [22].

Numerous approaches have been used for evaluating hepatic steatosis by computed tomography; the most important of which is determining the liver’s attenuation value. In the non-enhanced phase, the region of interest is set in the right hepatic lobe; if it is less than 40 HU, this indicates moderate hepatic steatosis with a fat liver percentage greater than 30% [23, 24]. Another way for assessing hepatic steatosis is to compare the area of interest in the splenic parenchyma to the liver, when we find the attenuation of liver is at least 10 HU less than that of the spleen. Several studies have demonstrated that non-enhanced CT has a great sensitivity (from 43 to 95%) and a great specificity (from 90 to 100%) for detecting hepatic steatosis [25–27].

## Methods

This retrospective study was conducted at our institution’s Radiology Department from May 1, 2020, to June 1, 2020. Approval was acquired from the Institution’s Ethics and Research Committee. Informed consent was taken.

### Inclusion criteria

Our research included 355 subjects who presented with flu-like symptoms and were suspected of being infected with COVID-19. They underwent PCR checking and chest CT for COVID-19. For all, we utilize the same 64-slice CT scanner (Siemens Healthcare, Germany).

The case group included 158 subjects (PCR positive for COVID-19), while the control group consisted of 197 subjects with a negative PCR test. It is widely established that false-negative RT-PCR can happen in infected individuals, but CT chest may reveal disease signs (positive CT). Therefore, to ensure the control group’s negativity, we checked their CT chest and retained only those who had a negative CT chest (PCR-negative and chest CT-negative pattern).

Two radiologists with over 10 years of expertise interpreted the CT chest.

CT evaluation involved identifying the areas of ground-glass opacities, crazy-paving patterns (ground-glass opacities with interlobular septal thickening), atelectatic bands, and consolidations. CT results were divided into five classes using the RSNA Expert Consensus Criteria [28], as well as the COVID-19 Reporting and Data System (CO-RADs) from the COVID-19 Working Group of the Dutch Radiological Society [29]. CT results are graded according to these grading methods as normal, inconsistent, or typical of COVID-19 pneumonia. The severity of lung affection (CT severity index) was measured as per Yang et al. A CT score of more than 20/40 indicates serious illness and is typically related to a poor prognosis [30].

We assess hepatic steatosis in our research by determining the attenuation of liver value. The area of interest (with an average area of 10 cm^2^) was located in the right hepatic lobe (between segments VI and VII), chosen area away from the biliary tree, vessels, or focal lesions. We examine one slice and define the liver as fatty if the HU reading is less than 40.

### Statistical analysis

SPSS (Statistical Package for the Social Sciences; SPSS Inc., Chicago, IL, USA) release 25 was utilized for all statistical calculations. Standard deviations and means are used to describe quantitative data, while percentages are used to indicate qualitative data. Per the variable distribution, we employed the t-Student and chi-square checks. A p-value below 0.05 was considered significant.

## Results

### Characteristics of study group (Table 1)

- Our research includes 355 subjects, 158 with positive PCR for COVID-19 (case group) and 197 with negative PCR and negative CT chest (control group). The mean age in the positive group was 50.6 ± 16 years, and in the control, it was 41.3 ± 16 years (p < 0.001).
- Our study comprised 321 men (90.5%) and 34 women (9.5%). The number of males in both cases and control groups was greater. In the case group, 93% men vs. 6.9% women, while in controls, 88.3% men vs.11.6% women, p < 0.001.
- CT demonstrated normal results in 55.5% of individuals (i.e., CORADs 1) and abnormal findings in 45.5% of participants (i.e., CORADs 2–5). In abnormal scan, CO-RADs 2 was 13.92%, while CO-RADs 3–4 were 20.89% of cases. CO-RADs 5 comprised 65.19% of all cases. Approximately 42.6% of cases had severe disease (CT score ≥ 20); all of them were CO-RADs 5.

**Table 1:** Demographic comparison parameters and statistics between the two groups

Parameter	Case group Positive PCR N = 158	Control group Negative PCR/negative chest CT N = 197
Age	50.6 ± 16 years	41.3 ± 16 years
Sex		
Male	N = 147 (93%)	N = 174 (88.3%)
Female	N = 11 (6.9%**)**	N = 23 (11.6%)
Steatosis	28.5%	12.2%
HU	46.79 ± 12.68	53.34 ± 10.28
CO-RADs	CO-RADs 2, N = 22 (13.92%)	CO-RADs 1, N = 197 (55.5%)
	CO-RADs 3–4, N = 33 (20.89%)	
	CO-RADs 5, N = 103 (65.19%)	

### Association with steatosis (Figs. 1, 2, 3, 4, and 5)

- The PCR-positive group had a greater prevalence of hepatic steatosis than controls (28.5% vs.12.2%, p < 0.001). CO-RADs 2 represented 11.1%, CO-RADs 3–4 represented 15.6%, and CO-RADs 5 represented 73.3% in the hepatic steatosis cases.
- The mean hepatic attenuation value in the case group was 46.79 ± 12.68, and in the control group, 53.34 ± 10.28 (p < 0.001).
- When comparing patients with a higher severity score (CT score ≥ 20) to those with non-severe pneumonia, it was discovered that hepatic steatosis is more prevalent (73.2% vs. 26.8%).

**Fig. 1:**
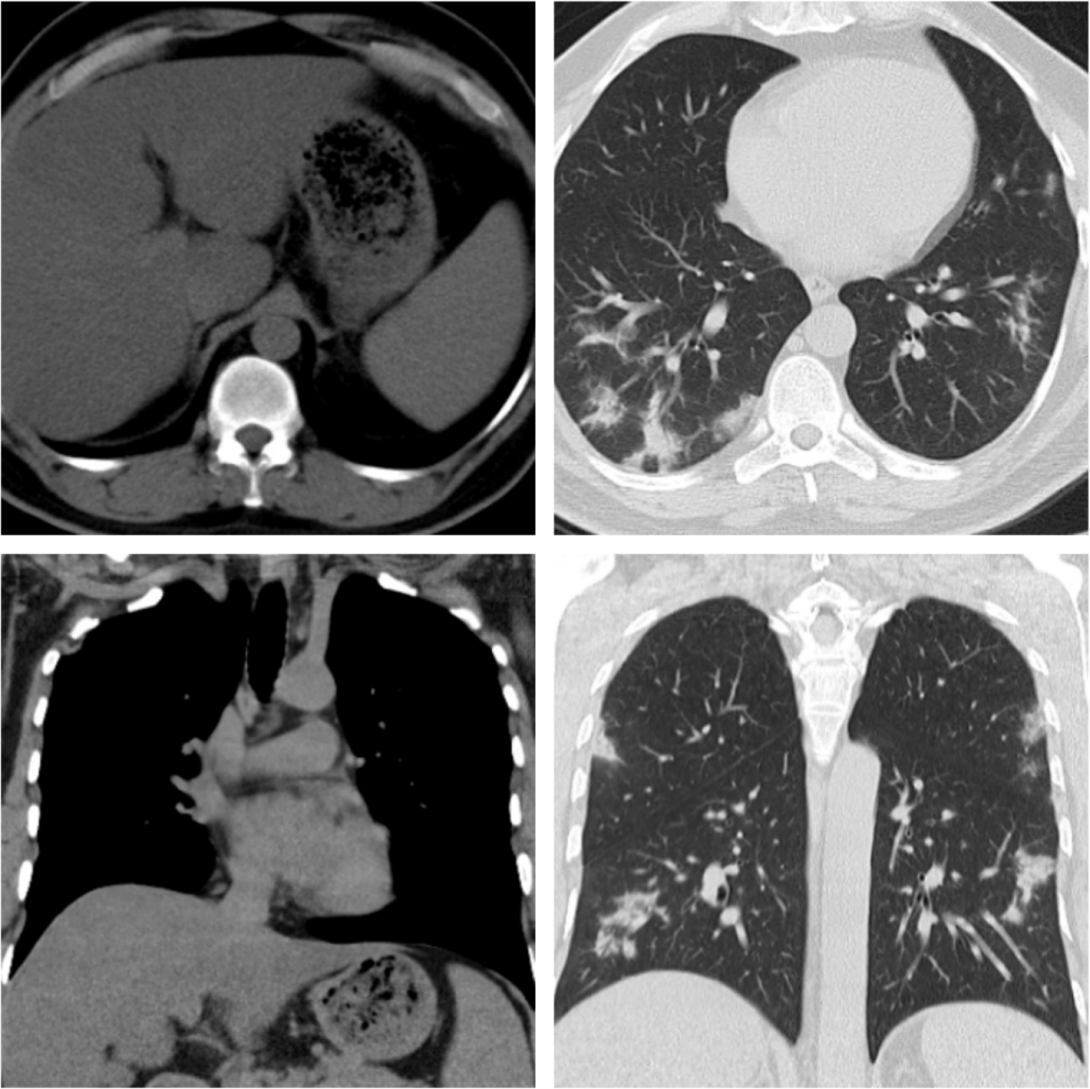
Male patient, 45 years old. CT chest shows multiple bilateral pulmonary patchy ground-glass opacities, reported as CORADs 5. Upper abdominal cuts show hepatic steatosis

**Fig. 2:**
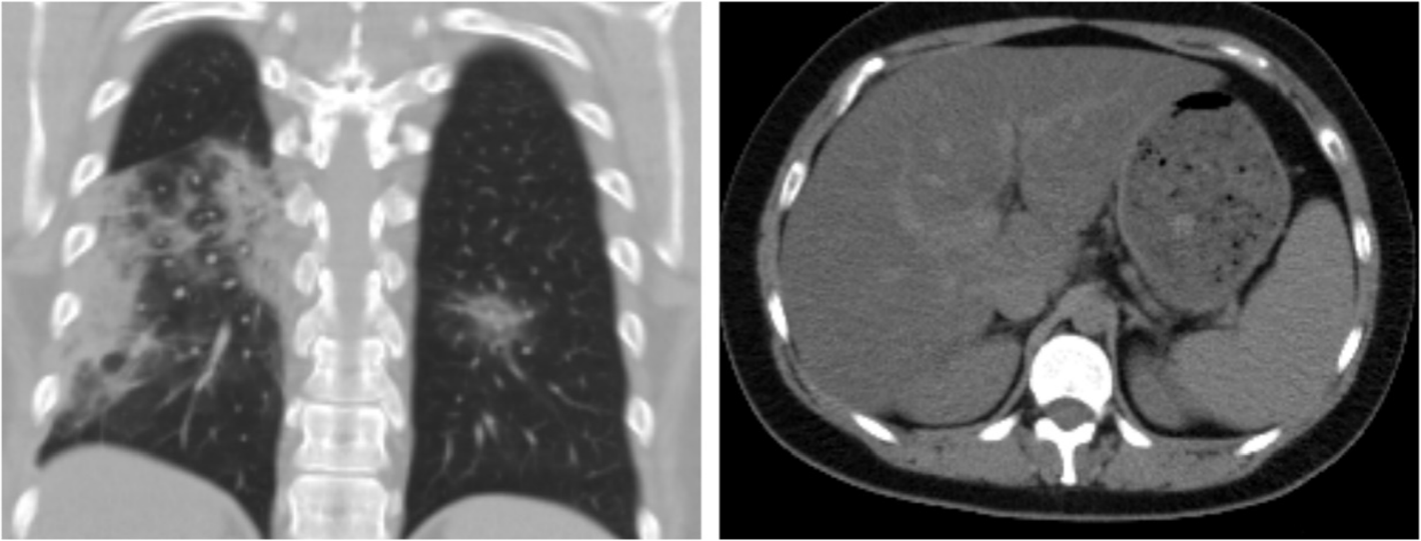
Female patient, 28 years old. CT chest shows multiple pulmonary ground-glass opacities mounting to consolidative patches, reported as CORADs 5. Upper abdominal cuts show diffuse low parenchymal attenuation of the liver denoting fatty infiltration

**Fig. 3:**
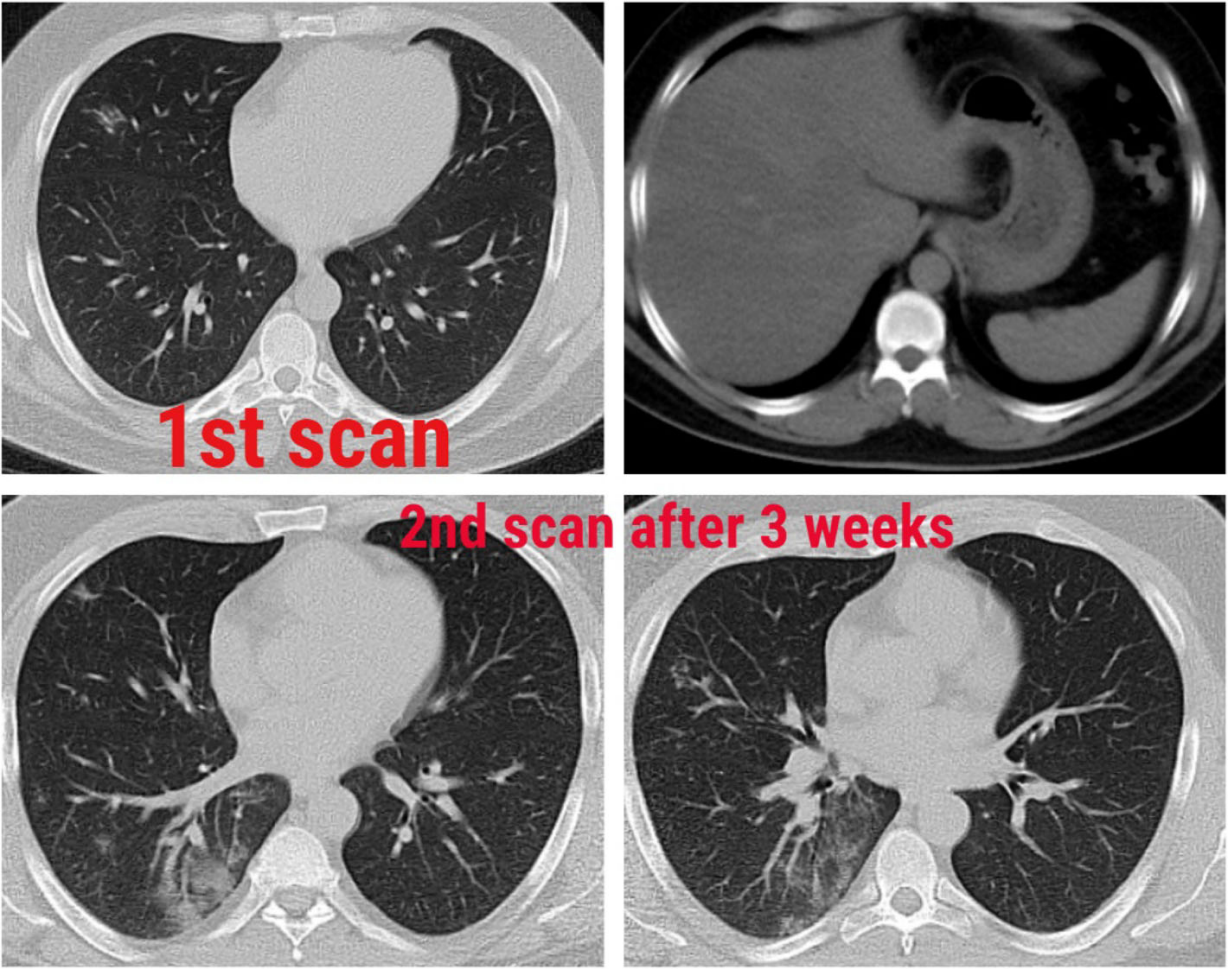
Male patient, 36 years old. The first scan after few days of symptoms shows just a small patchy ground-glass opacity in the middle lobe. The second scan after 3 weeks for follow-up shows a progressive course with multiple pulmonary ground-glass opacities. CT cuts of the upper abdomen show a fatty liver

**Fig. 4:**
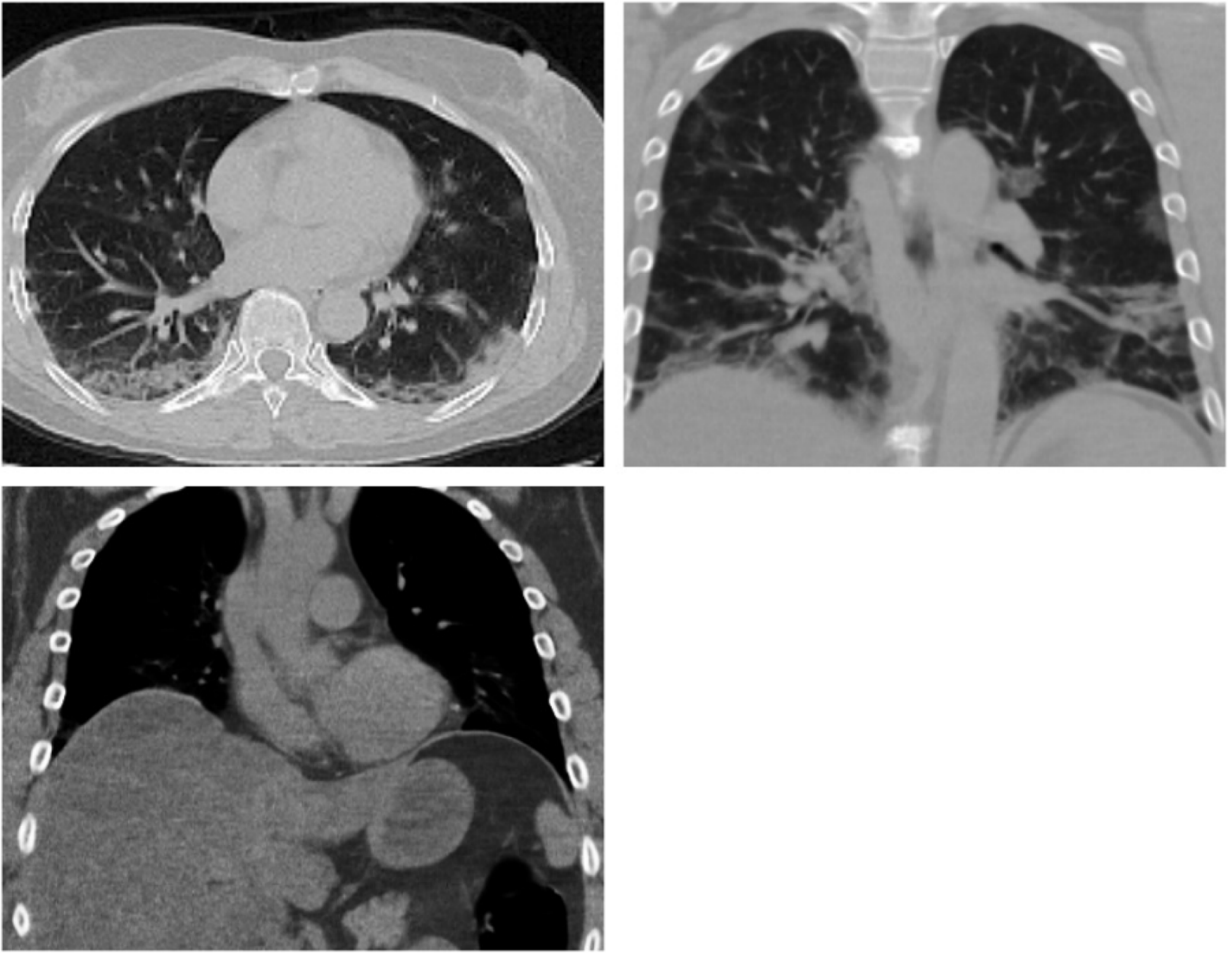
Male patient, 64 years. CT chest shows bilateral lower lobar subpleural patchy ground-glass opacities with underlying interlobular septal thickening and atelectatic bands, reported as CORADs 5. Upper abdominal cuts show fatty hepatomegaly

**Fig. 5:**
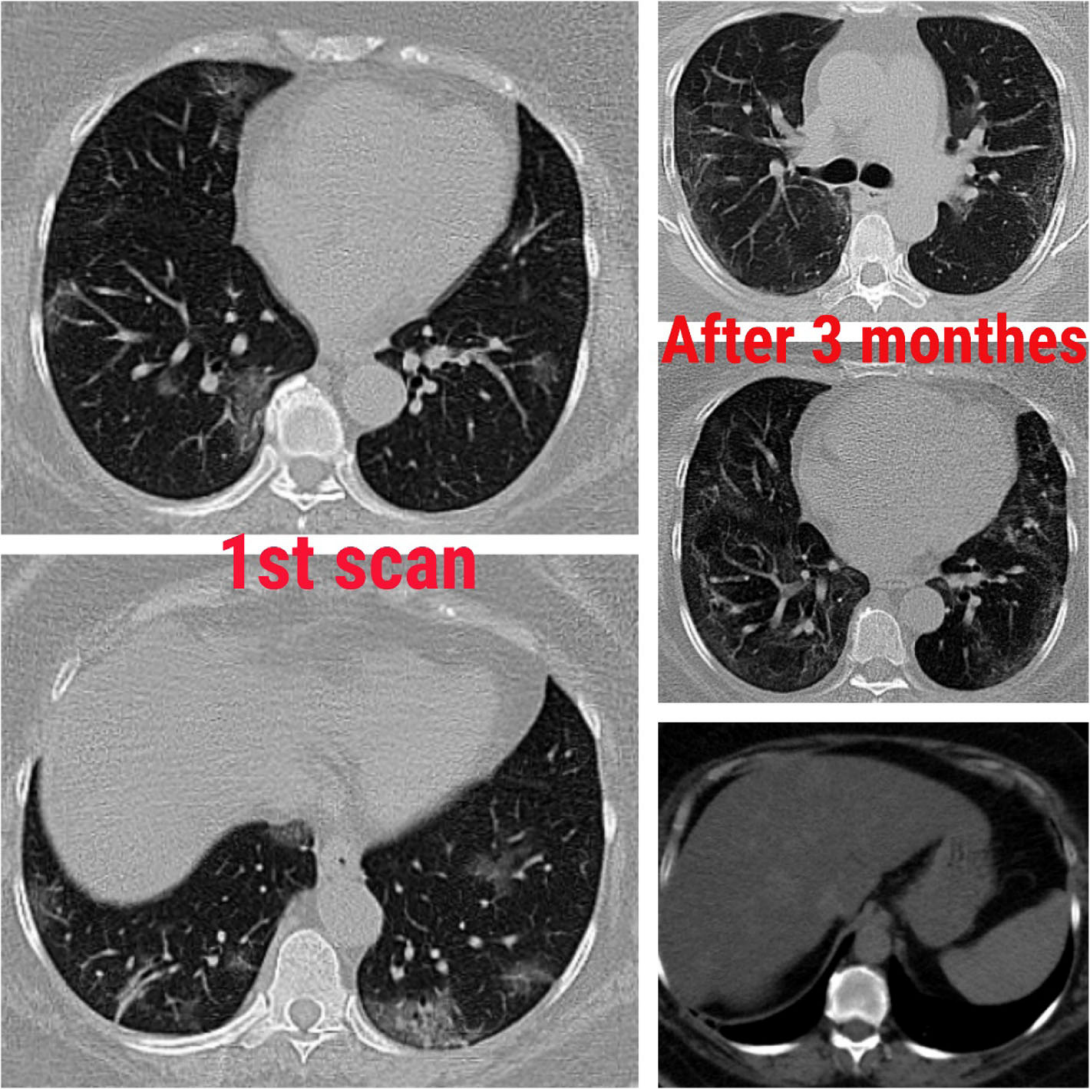
Female patient, 58 years old. The first scan shows multiple subpleural ground-glass opacities on follow-up after 3 months. CT shows multiple subpleural parenchymal bands. Upper abdominal cuts show fatty hepatomegaly

## Discussion

In 2016, the World Health Organization reported that 1.9 billion adults were overweight, with over 650 million being obese [31]. Obesity is the most major and significant risk factor in developing hepatic steatosis in adults and children [32].

Obesity is thought to be a condition of low-grade systemic inflammation that has been linked to a variety of metabolic diseases like type 2 diabetes mellitus and dyslipidemia. It can alter immunological responses, causing the immune system more sensitive to infection development [33].

Due to its endocrine roles and the release of various adipokines and proinflammatory cytokines like leptin, interleukin 6, C-reactive protein, visceral adipose tissue, and TNF are more metabolically active than subcutaneous adipose tissue [34, 35]. It is well documented that raised IL-6 levels are related to chronic inflammatory airway disorder. Numerous studies have discovered greater IL-6 concentrations in post-mortem specimens from COVID-19 cases [36, 37]. Leptin has been linked with airway reactivity, and current research indicates that leptin concentrations are increased in COVID-19 cases with significant pulmonary inflammation [38, 39].

COVID-19 invades human cells through binding with angiotensin-converting enzyme 2, and some research shows that the renin-angiotensin-aldosterone system’s imbalanced activity in obese individuals contributes to this pathogenesis. Because ACE2 expression is greater in adipose tissue than in lung tissue, and because ACE2 in lung tissue is known to be the primary entry point for SARS-CoV-2, this increases the sensitivity of obese patients to infection [40].

Additionally, obese individuals have impaired B and T cell responses due to changes in the quantity and function of lymphocytes, resulting in an increased vulnerability to viral infection. In virally infected obese individuals, the inflammatory response is dysregulated, resulting in a reduction and delay in macrophage activation [41]. Obesity can promote antiviral resistance as well [42].

NLR, a measure of systemic inflammation, was considerably elevated and related to poorer results in cases infected with COVID-19 [43]. There is a substantial correlation between this ratio and the severity of liver fibrosis in people with NAFLD. Current research has established that this association affects the COVID-19-induced inflammatory storm, which is associated with raised death and morbidity. In cases infected with COVID-19, liver injury occurs following lung injury [44]. This destruction could be caused by the overactivation of Kupffer cells, the production of a cytotoxic T cell response produced by the virus, or the production of a dysregulated innate immune response [45]. In these cases, post-mortem liver biopsies revealed microvascular steatosis [44].

According to Zheng et al., individuals with metabolic-associated fatty liver disease and obesity had a sixfold raised chance of developing severe COVID-19 infection [46]. Another research indicated that populations with metabolically related fatty liver disease (MAFLD) have a fourfold greater risk of developing severe forms of COVID-19 [47].

Per Palomar-Lever et al.’s findings, the combination of obesity and hepatic steatosis led to a significant relationship with serious illness, implying a synergic connection between both [20].

Medeiros et al. concluded that the steatosis prevalence on CT was greater in confirmed COVID pneumonia cases than in the control. This is important for radiologists because liver steatosis can be easily assessed and verified by any radiologist reading a chest CT. Moreover, this data can be added to the clinical data available to clinicians [48].

In research conducted in New York, cases with a body mass index (BMI) of ≥ 30 had a higher chance of acute care hospitalization, and those with a BMI of ≥ 35 had a higher risk of intensive care unit admission [49].

Additionally, it was observed that cases with NAFLD had a greater rate of progression to severe illness and poorer findings in COVID19 [44, 50, 51].

Ji et al. studied NAFLD in 202 cases with COVID-19 using the hepatic steatosis index based on ALT, AST, body mass index, gender, presence of diabetes, and/or an ultrasound examination. They discovered that preexisting comorbidities and NAFLD were linked with COVID-19 progression [44].

According to Zhou et al., the risk of severe COVID-19 increases fourfold when metabolic-related fatty liver disease is present [52].

As with the previous research, univariate and multivariate analyses suggested that individuals with NAFLD had an increased risk of disease progression. Comorbidities like diabetes mellitus, hypertension, coronary artery disease, and COPD are identified as additional risk parameters for COVID-19 progression [44, 53].

Petersen et al. used low-dose computed tomography and post-processing software to measure body fat distribution particularly visceral adipose tissue and upper abdominal circumference in COVID patients and found that these two parameters significantly increase the likelihood of COVID-19 severe courses [54].

Parlak et al. found that chest CT, which is critical for diagnosing COVID-19, can provide data about the disease’s prognosis and that fatty liver is a significant indicator of a bad prognosis and may be easily spotted on chest CT used for COVID-19 diagnosis [55].

### Limitations

Other significant variables like hypertension, lipid profile, diabetes, weight, obesity, body mass index, and liver function were not evaluated. Hence, a correlation between these variables and hepatic steatosis could not be established.

## Conclusion

In confirmed COVID-19 cases, our research demonstrates a considerably greater frequency of hepatic steatosis by CT as compared to controls. There is a correlation among metabolic syndrome, steatosis of the liver, and obesity, as well as the severity of COVID-19.

## Data Availability

The datasets used and analyzed during the current study are available from the corresponding author on reasonable request.

## Abbreviations

RT-PCR: Real-time reverse transcription polymerase chain reaction
COVID-19: Coronavirus disease of 2019
MAFLD: Metabolic fatty liver disease
NLR: Neutrophil-to-lymphocyte ratio
NAFLD: Non-alcoholic fatty liver disease
IL-6: Interleukin 6
HU: Hounsfield unit
SARS-CoV-2: Severe acute respiratory syndrome coronavirus 2
ACE2: Angiotensin-converting enzyme 2
HS: Hepatic steatosis
